# Credentialing for radiology

**DOI:** 10.2349/biij.4.1.e14

**Published:** 2008-01-01

**Authors:** M Street, KR Thomson

**Affiliations:** 1 Radiology Department, The Alfred Hospital, Melbourne, Victoria, Australia; 2 Department of Medicine, Monash University, The Alfred Hospital, Melbourne, Victoria, Australia

**Keywords:** Credentialing, certification, radiology, interventional, accreditation

## Abstract

Patients expect to receive safe, predictable and high-quality care delivered by competent professionals. Thus, it has become important to provide specific training in existing and new modalities and prove on-going clinical expertise. Hospital credentialing is the process by which the competence of a doctor is determined by the hospital management. In Australia, radiologists participate in a mandatory program of continuing professional development and are also required to maintain a logbook of procedures. The *Conjoint Committee for the Recognition of Training in Peripheral Endovascular Therapy* has been established to advise the respective subspecialty groups on the requirements for accreditation. This article examines some of the issues the committee has considered in preparing the criteria to assist institutions for the purposes of credentialing and gives an Australian perspective on future trends.

## CREDENTIALING FOR RADIOLOGY

Recent events in Australia, where a surgeon was found to be inadequately qualified to treat patients safely [1, 2], have increased the awareness of the general public and hospital administrators to the need for adequate credentialing of medical doctors, particularly those performing operative procedures.

There is an expectation that patients will receive safe, predictable and high-quality care delivered by competent professionals [3]. Due to the rapid development and expansion of diagnostic and interventional radiology over the past 20 years, it has become important to provide specific training in existing and new modalities. However it is equally crucial to prove initial and on-going capacity to deliver a safe service for the patient. This capability requires clinical expertise and a commitment to the process of continuous education [4].

Hospital credentialing is the process by which the competence of a doctor is determined by the hospital management [5]. With appropriate credentials, a medical practitioner can then be accredited for practice in the areas of work for which the credentials cover. Often these two processes are confused. Accreditation is achieved through documentation of a proven course of training, performance of the procedure within recognised and accepted norms, and most importantly, continued competency in the performance of the procedure. While professional organisations provide opportunities for continuing education, they do not provide credentialling; places of work must do this. Clearly the ultimate goal is to achieve ongoing improvement of practice and to demonstrate competency as a medical practitioner [3].

In Australia, radiologists who are Fellows of the Royal Australian and New Zealand College of Radiologists participate in a mandatory program of continuing professional development (CPD) organised and audited by the College. CPD provides the opportunity for Fellows to engage in activities relevant to their professional development, educationally and in other ways. It helps them to maintain their skills and knowledge in their chosen discipline. It provides an opportunity for them to contribute to the profession through engagement with others [6].

### CPD Points Requirements

One CPD point is approximately equivalent to one hour of passive education (e.g. attending a lecture). In general, active educational activities are allocated 2 CPD points per hour; case-based activities are allocated points on a 'per case' basis, while complex activities (such as audit) are allocated points on a 'per activity' basis.

- The CPD program operates on a calendar year – i.e. from 1 January to 31 December of each year.
- Participants should accumulate a minimum of 180 points in the triennium (currently 2007-2009).
- Participants should accumulate a minimum of 30 points per CPD year, while no more than 90 points will be credited to any one year.

Participants should also aim to acquire their points across a range of categories, which include medical expert, communicator, collaborator, health advocate, manager, professional and scholar, to give an indication of the major emphasis on the capabilities being developed in the CPD activity group. Participants can also complete their CPD returns on-line.

In addition, interventional radiologists are required to maintain a logbook of procedures including the complications and outcome faced by the patient. The Radiological Percutaneous Interventional Database (RaPID) is an electronic database available by registration through the Interventional Radiology Society of Australasia (IRSA) [7]. From 2008 these processes, which were originally voluntary, have become mandatory. They are subject to random audit by the Royal Australian and New Zealand College of Radiologists.

At the hospital level it is recognised that due to the complexity of modern radiology a single radiologist may not have the necessary experience and expertise in every imaging modality or procedure. As a consequence, clinical privileges are only granted with evidence of proof of adequate training, expertise and documented performance. This has led to the development of guidelines for both training and competency. While the requirements for new graduates are relatively straightforward, it is important that the experience of older graduates be recognised. Thus the ‘grandfather’ qualification has been introduced to demonstrate that an individual practitioner has sufficient experience and competence [8]. In most cases when a new modality or procedure is developed, it is necessary to determine what experience and proof is required for ’grandfathering’ older specialists.

In the proposed national registration requirements for specialist radiologists, the Commonwealth Government of Australia has asked for input from radiologists and other specialists including cardiologists and vascular surgeons who perform aspects of interventional radiology. This has resulted in the formation of the *Conjoint Committee for the Recognition of Training in Peripheral Endovascular Therapy* (CCoPET). This committee is a joint initiative of the Royal Australasian College of Surgeons, the Royal Australasian College of Physicians and the Royal Australian & New Zealand College of Radiologists [9]. This Committee has been established to advise the specialist colleges of appropriate criteria for training of peripheral endovascular therapists who wish to practice in Australia or New Zealand. The criteria established by the Conjoint Committee are then available to institutions for the purposes of credentialing. Each subspecialty has representatives on the committee and decisions regarding the extent of training are made by consensus. This committee does not provide any certification or examination; it merely serves to advise the respective subspecialty groups on the requirements for accreditation.

The greatest challenge facing committees of this type is determining what kind of training is required and how many procedures are needed to demonstrate competency for new graduates and ongoing accreditation. In radiology there is competition from specialists of different disciplines for the same procedure [10, 11]. It is extremely important that the lofty ideals of credentialing are not used as a weapon to exclude suitably qualified medical practitioners from practicing their craft. One example would be if the same requirements were applied to cervical (extra cranial) carotid artery stenting as to the more rigorous procedures for intracranial interventions and acute stroke intervention [4].

However, each subspecialty has its own idea of how long training needs to be. Radiologists are generally surprised that extensive training in all CV imaging modalities can be achieved in a single year of training as suggested by the American College of Cardiology [12]. By contrast, does every imaging specialist need 5 years of general radiology and barium enema experience to be a skilled interventional radiologist? The reality in Australia is that additional fellowship training is required for interventional radiology.

Some of the suggested requirements for accreditation are becoming difficult to achieve due to changes in clinical guidelines and practice. For example an accumulated total of 100 diagnostic cervicocerebral angiograms before postgraduate training in coronary artery stenting procedures [12] ignores the rapid displacement of cervicocerebral angiograms by other imaging modalities especially CTA and MRA.

Another problem which the Conjoint Committee has faced is in determining the number of procedures required for unusual or rarely performed procedures. In this instance the concept of equivalency of more commonly performed procedures has been used to indicate competency in a more general way. For example a doctor who has completed many angioplasty procedures may be considered competent to perform selective thrombolysis. While not ideal, this is particularly relevant for practitioners in remote areas or in small hospitals with limited numbers of procedures, who may struggle to achieve the required numbers to prove competency. One solution may be to provide access for such persons in larger centres to undertake training from time to time. This would need to be supported by providing locum services for their remote practices during these training periods. However, remote area practitioners would then be competing for cases with new trainees. The alternative of a “remote area exemption”, such as applies in respect to radiologist attendance in some types of musculo-skeletal ultrasound, would not be appropriate for credentialing.

Credentialing of diagnostic radiology is simpler than for interventional radiology procedures. Patient selection, informed consent and technical procedural skills are not generally required by diagnostic radiologists.

One solution, which is widespread in screening mammography, is double reading to improve sensitivity and accuracy. Computer-aided detection is also used to reduce the human costs involved in double reading [13]. However, these practices are not easily transferable to a busy general radiology practice. With more widespread use of PACS it will be possible to provide random audits of previous reports and possibly also document outcomes. However, outcome analysis is not generally possible in a small clinical radiology service, as patients with more complex conditions may be transferred.

## FUTURE TRENDS

It is becoming increasingly difficult to learn interventional radiology skills because of fewer “straightforward” procedures and growing concerns for patient safety [14]. Computer-based simulation has the potential to allow an operator to realistically perform a virtual procedure with feedback about performance, which could at least reduce some of the patient's role during the learning process [14]. The requirements for outcome-based and proficiency-based assessments have increased interest in the use of simulators for interventional radiological procedures. While they cannot replicate the experience of performing cases in real patients, there may be a role for it in procedural training in the future [15].

Radiologists need to maintain certification and documentation of professional competency. This ensures on-going knowledge of new advances in the field and up-to-date methods. In the future the task of auditing might be tendered to a large academic institution and the results of the audit benchmarked across several institutions. Because of the sensitivities involved, however, such an audit process is still some way off.

Given the cost and potential risk of interventional radiology, it is inevitable that institutions and governments will develop their own set of regulations for the practice of radiology, unless subspecialties provide suitable alternatives. In the meantime, each radiologist should personally consider how well-qualified they are to perform the tasks they currently undertake and how they would be able to prove their safety and competency. While some may find this an onerous task, ultimately it is the patients who will benefit.
